# Nitrogen Distribution and Cycling through Water Flows in a Subtropical Bamboo Forest under High Level of Atmospheric Deposition

**DOI:** 10.1371/journal.pone.0075862

**Published:** 2013-10-11

**Authors:** Li-hua Tu, Ting-xing Hu, Jian Zhang, Li-hua Huang, Yin-long Xiao, Gang Chen, Hong-ling Hu, Li Liu, Jiang-kun Zheng, Zhen-feng Xu, Liang-hua Chen

**Affiliations:** 1 College of Forestry, Sichuan Agricultural University, Ya'an, Sichuan, China; 2 Institute of Ecological Forestry, College of Forestry, Sichuan Agricultural University Chengdu Campus, Chengdu, China; 3 Maize Research Institute, Sichuan Agricultural University Chengdu Campus, Chengdu, China; 4 Personnel Department, Sichuan Agricultural University, Ya'an, Sichuan, China; Roehampton University, United Kingdom

## Abstract

**Background:**

The hydrological cycle is an important way of transportation and reallocation of reactive nitrogen (N) in forest ecosystems. However, under a high level of atmospheric N deposition, the N distribution and cycling through water flows in forest ecosystems especially in bamboo ecosystems are not well understood.

**Methodology/Principal Findings:**

In order to investigate N fluxes through water flows in a *Pleioblastus amarus* bamboo forest, event rainfall/snowfall (precipitation, PP), throughfall (TF), stemflow (SF), surface runoff (SR), forest floor leachate (FFL), soil water at the depth of 40 cm (SW1) and 100 cm (SW2) were collected and measured through the whole year of 2009. Nitrogen distribution in different pools in this ecosystem was also measured. Mean N pools in vegetation and soil (0–1 m) were 351.7 and 7752.8 kg ha^−1^. Open field nitrogen deposition at the study site was 113.8 kg N ha^−1^ yr^−1^, which was one of the highest in the world. N-NH_4_^+^, N-NO_3_^−^ and dissolved organic N (DON) accounted for 54%, 22% and 24% of total wet N deposition. Net canopy accumulated of N occurred with N-NO_3_^−^ and DON but not N-NH_4_^+^. The flux of total dissolved N (TDN) to the forest floor was greater than that in open field precipitation by 17.7 kg N ha^−1^ yr^−1^, due to capture of dry and cloudwater deposition net of canopy uptake. There were significant negative exponential relationships between monthly water flow depths and monthly mean TDN concentrations in PP, TF, SR, FFL and SW1.

**Conclusions/Significance:**

The open field nitrogen deposition through precipitation is very high over the world, which is the main way of reactive N input in this bamboo ecosystem. The water exchange and N consume mainly occurred in the litter floor layer and topsoil layer, where most of fine roots of bamboo distributed.

## Introduction

Nitrogen (N) is a fundamental element for living organisms, and the global N cycle has been strongly influenced by human activity. Between 1860 and the early 1990s, the amount of reactive N created by anthropogenic processes increased rapidly, which exceeds the reactive N created by natural terrestrial processes [1]. At the end of 20^th^ century, human activities became the dominant force in the transformation of N_2_ to reactive N on land [1]. Global industrial emissions of NO_x_ and agricultural emissions of NH_3_ into the atmosphere were 52.1 and 64.6 Tg N yr^−1^ in 2000, respectively [2]. Large amounts of NO_x_ and NH_3_ emitted into the atmosphere can be converted into a series of compounds and ultimately deposited in terrestrial and aquatic ecosystems (N deposition) [2]. It is predicted that emissions of NO_x_ and NH_3_ with consequent N deposition will increase considerably in the 21^st^ century, especially in East and South Asia. Conversely, N deposition in many areas in Europe and some parts of North America is going down [1]–[3].

Increased atmospheric N deposition and consequent ecological impacts on forest ecosystems in the temperate and boreal zones of Europe and North America have been well documented (e.g., special issues of Forest Ecology Management, 196[2004]; Högberg [4], de Vries et al. [5] and Dezi et al. [6]). For example, the response of plant growth to N deposition is generally positive in temperate and boreal forest ecosystems [5], [7]; elevated NO_3_^−^ leaching appear in some forests when the N deposition above 10 kg ha^−1^ yr^−1^ and always above 25 kg ha^−1^ yr^−1^ [8].

Hydrological process in forest is composed of many important water flow paths, such as precipitation (PP), throughfall (TF), stemflow (SF), surface runoff (SR), forest floor leachate (FFL), and soil water. Throughfall is the precipitation that passes through the forest canopy, either directly through the gaps or by interacting with the vegetation [9]. Stemflow is the precipitation that is funneled down the trunks of the trees [10]. Surface runoff, forest floor leachate and soil water are the reallocation of water inputs to the forest surface. All of these processes greatly affect water allocation and nutrient cycling. The water fluxes are a major control of the dynamics of inorganic N [11] and dissolved organic N (DON) [12], [13] in forest ecosystems, especially after rainstorms. Throughfall [14]–[16] or TF plus SF [17] fluxes of N are often used as a first approximation of the total N deposition in forest ecosystems.

Bamboo forests are one of the most important and fastest growing forest types in the world, and they are mainly distributed in the tropical and subtropical zones [18]. Bamboo species account for approximately 0.8% of the world's total forested area [18]. China is one of the distribution centers of bamboo species, and China's bamboo forests covered an area of 4.84 million ha in 2005, accounting for 15.4% of the total area of bamboo worldwide [18], [19]. Although many studies have examined the N fluxes through water flows in natural and plantation forests [16], [20]–[23], no one study has assessed these processes in bamboo forests, and only a few studies (e.g., Onozawa et al. [24]) have studied TF and rainfall interception in bamboo ecosystems.

The aims of this study were 1) quantify the N input through atmospheric N deposition, and N cycling through water flow paths – PP, TF, SF, SR, FFL, soil water at the depth of 40 cm (SW1) and 100 cm (SW2) in a subtropical bamboo forest; 2) quantify the N pools distribution among plant, litter floor and soil; 3) investigate the N dynamics in different water flows in this ecosystem.

## Materials and Methods

### Site description

The study was conducted in a *Pleioblastus amarus* plantation in Liujiang, Sichuan, China (29°42′N, 103°14′E), with an about 600 m a.s.l. elevation and a mid-subtropical, humid, mountainous climate [25]. The research site is owned by collective organization of Liupa village, Liujiang town, Sichuan, China. The field studies did not involve endangered or protected species and no specific permits were required for the described field studies.

The annual mean relative humidity was 86%, and the monthly mean temperature is 6.6°C in January and 25.7°C in July. The mean annual precipitation from 1980 to 2000 was 1490 mm. The annual frost-free period is 352–360 days. In this region, the precipitation frequency is very high, thus soil moisture is almost stable (30–45 cm^3^ H_2_O 100 cm^−3^ in the 0–15 cm soil horizon) throughout the year [26].

The site (10 ha) was converted from cropland to a *Pleioblastus amarus* plantation in 2000 as part of the National Project of Converting Farmland to Forests (NPCFF). There is a wide planting area of *P. amarus*, a major bamboo species in the NPCFF. The canopy density is about 0.9. The mean density of the study stand was 35 354 stems ha^−1^ in November 2008, and the average stem height was about 7.5 m. Diameters at breast height (DBH, 1.3 m above the ground) was distributed between <2.0 cm, 2.1–4.0 cm, and >4.0 cm, which accounted for 7%, 78%, and 15%, respectively, of the forest. The forest floor litter accumulation was 2.0 Mg ha^−1^. The soil at the site belongs to Dystric Purpli-Orthic Primosols (Soil Survey USDA—Order: Inceptisols; Suborder: Udepts; Great group: Dystrudepts; Subgroup: Lithic Dystrudepts) and is derived from purple sandstone and shale [27], with depth about 100 cm and pH around 4.6. There was very little shrubbery or grass in the understory at the time of the experiment.

In December 2008, three 30 m×30 m (0.09 ha) N cycling measurement plots at >20 m intervals were established, close to the subplots used for the already-reported studies of carbon sequestration [26], litter decomposition [28] and soil respiration [29] under simulated N deposition. The slope aspect is south, and the slope degree is about 15° for all three plots.

### Nitrogen pool measurements

Diameters at breast height (DBH) and stem height values were recorded on all living stems in each plot in December 2008. The biomass of different parts (foliage, branch, bole wood, and rhizome) were estimated using biomass models for the *P. amarus* plantation [30] (models' details were stated in *Data analysis*). The root biomass was determined by collecting soil samples in December 2008, using a *Φ*15 cm×40 cm corer. Twelve core samples were randomly collected from each plot. Roots were weighed after separation by washing, dried at 85°C for 48 h, and weighed. Five samples of forest floor were collected using a 0.5 m×0.5 m frame in each plot. Samples were dried at 85°C for 48 h, and weighed. And then, each sample of the root and forest floor was ground using a Wiley mill with a 1 mm mesh screen, and stored in a paper bag for chemical analysis. Fifteen trees were randomly selected and cut down in the experimental site but outside of the three N cycling measurement plots. Samples of foliage, branch, bole and rhizome were collected, dried at 85°C until constant, ground with a 1 mm mesh screen, and stored for analysis. The mineral soil was collected at the following depths: 0–5, 5–20, 20–40, 40–60, 60–80, and 80–100 cm using a 5-cm diameter auger. Five 5 cm×100 cm soil cores from each plot were collected. For each plot, composite samples by soil layer were gently mixed, air dried, the visible roots were removed by tweezers, and then the soil sample was homogenized, grinded and passed through a 0.3 mm sieve and stored in a paper bag for analysis. The litter in five 50 cm×50 cm subplots was combined into one sample (three in total). All the tissue and litter samples were dried at 65°C for 48 h, ground using a Wiley mill with a 1-mm mesh screen, and stored in a paper bag for analysis.

### Monitoring nitrogen flux through water flows

1) Precipitation (PP) and throughfall (TF). The continuous 10-min PP intensity and hourly temperature data were recorded by a Vantage Pro Weather Station (Davis Inc. USA) placed on a pasture about 300 m from the forest. In addition, event PP samples were collected with six trough-type collectors from January 1 to December 31, 2009. The collectors, installed on supports 1 m above the ground, were made from 1-m length and 200-mm diameter PVC pipes (the horizontal area is 0.2 m^2^), which were connected via flexible tubes to 40-l plastic canisters. Each collector was covered by 1-mm nylon mesh to prevent plant tissue debris, insects and other materials from entering the collectors. The nylon meshes were cleaned regularly to avoid blockages. Nine collectors (the same specifications as the PP collectors) were placed in each plot to sample TF. Each plot was subdivided into nine subplots (10 m×10 m) and one collector was located within each subplot. Precipitation and TF volumes were collected for measurement of solute concentrations 2 h after every event or alternatively the next morning for events that ended after 09:00 pm.

2) Stemflow (SF). To measure SF volumes, representative samples of 18 stems were selected in each plot. Trees that were to be fitted with spiral SF collars were selected randomly and in proportion to the total number of stems in each diameter class in the plots (the average stem density of diameter classes <2.0, 2.0–4.0, and >4.0 cm DBH were 2376, 27755 and 6899 stems hm^−2^, therefore, 2, 12, and 4 stems in diameter classes <2.0, 2.0–4.0, and >4.0 cm DBH were chosen in each plot). Each SF collar (mounted 1.3 m above the forest floor) consisted of a 1-cm diameter rubber hose slit longitudinally and sealed to the trunk in an upward spiral and connected to a black 10-l plastic pot. Collars were checked regularly for leaks. The volume of SF for each tree was measured 2 h after every PP event and collected for chemical analysis.

3) Surface runoff (SR). Surface runoff was measured in a long-term SR observation site in the *P. amarus* plantation, established in 2000, adjacent to the N cycling plots (within 200 m). The SR observation site consisted of three 10×5 m runoff plots (with the same slope aspect and degree as the N cycling plots) and an observation room. The four sides of each plot were separated by a 10 cm wide cement ridge (40 cm in the soil and 30 cm above the ground). There was a sedimentation cistern in the observation room for each runoff plot. The SR depth was determined weekly, and then the samples were collected for chemical analysis.

4) Forest floor leachate (FFL). In each N cycling measurement plot, six FFL collectors were randomly placed under the litter layer. The collector was a 15×15×2 cm (length×width×height) plastic plate with an outlet pipe (1 cm in diameter) at the center and connected via a rubber tube to a 2-l plastic pot, which was placed in a 20×20×40 cm (length×width×depth) pit adjacent to the collector (within 1 m). The forest floor litter was generally about 1 cm thick before the experiment commenced. All the litter within the collector area (15×15 cm) was placed into the collector, which was laid on the mineral soil surface. The FFL collectors were checked regularly, and samples were collected for analysis.

5) Soil water. At each plot, six 1×1×1.2 m (length×width×depth) standard pits were dug and distributed in a straight line (perpendicular to the slope) across the middle of the plot at about 4 m intervals. Two soil water collectors were horizontally inserted into the upper soil profile, approximately 40 and 100 cm below the surface. The two collectors did not overlap vertically. The horizontal area of the aluminum sheet collector was 750 cm^2^ (30×25 cm). The dip of the collector was about 15°, and the outlet pipe was connected to a 2-l plastic pot by a rubber tube. The collectors were checked regularly, and the soil water samples at the 40 cm (SW1) and 100 cm (SW2) depths were collected regularly for analysis.

After each collection, the collectors were rinsed with deionized water. All the samples were collected in Nalgene polyethylene bottles that were pre-washed with diluted (5%) hydrochloric acid (HCl) and then thoroughly rinsed with deionized water. The samples were returned to the field laboratory and stored on ice in coolers until analysis.

### Litterfall measurements

Litterfall was measured in an adjacent 40×40 m plot. Ten 50 cm×50 cm litter traps were randomly located in the plot. Each 100 cm tall trap consisted of a metal hoop with a 50 cm deep, 1-mm mesh nylon netting bag. Organic material was collected every 15 days, separated into litter fractions (e.g., leaves, branches, sheathes), dried at 65°C to a constant mass for 48 h, and weighed. The oven-dried litter samples were ground using a Wiley mill with a 1-mm mesh screen and saved for chemical analysis.

### Chemical analysis

The N concentrations in tissue and soil samples were determined by acid digestion according to the Kjeldahl method [31]. A 200 mg sample was digested in 5 ml of 1.84 g ml^−1^ sulfuric acid and a 1.0 g mixture of potassium sulfate, copper sulfate, and selenium. The digestions were distilled using a UDK 142 automatic distillation unit (VELP, Milano, Italy).

After water samples returned to room temperature, pH was measured within 24 h using a PHS-3E glass pH electrode (Leichi Inc., China). After being filtered through polycarbonate membranes (0.45 µm, Whatman), the samples were stored in a freezer until analysis. Total dissolved N (TDN) was measured using a total C & N analyzer (TOC-V_cPH+TNM-1_, Shimazu Inc., Japan); N-NH_4_^+^ was determined by colorimetry using the Nessler's reagent method [32]; and N-NO_3_^−^ was measured based on delta-absorbance under UV light using a UV-1102 spectrophotometer (Tianmei Inc., China) at the wavelengths 220 and 275 nm. DON was calculated by the difference between TDN and the sum of N-NH_4_^+^ and N-NO_3_^−^.

### Data analysis

Biomass models of *P. amarus* [30]:
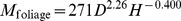


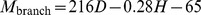


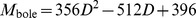


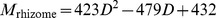

*M*_foliage_, *M*_branch_, *M*_bole_, *M*_rhizome_ represent dry biomass (g) of foliage, branch, bole and rhizome of *P. amarus*. *D* and *H* are DBH (1.3 m above the ground, cm) and stem height (m).

The sample stems of SF were grouped into size classes (<2.0 cm, 2.0–4.0 cm, and >4.0 cm DBH). Mean SF volumes were calculated for each class, and then multiplied by the number of trees in those size classes for each plot. The effects of forest vertical structure on N fluxes were calculated using the following equations:
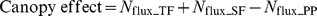


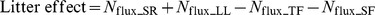


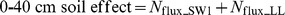


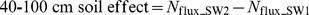



## Results

### Nitrogen in biomass, litterfall and soil

**Figure 1:**
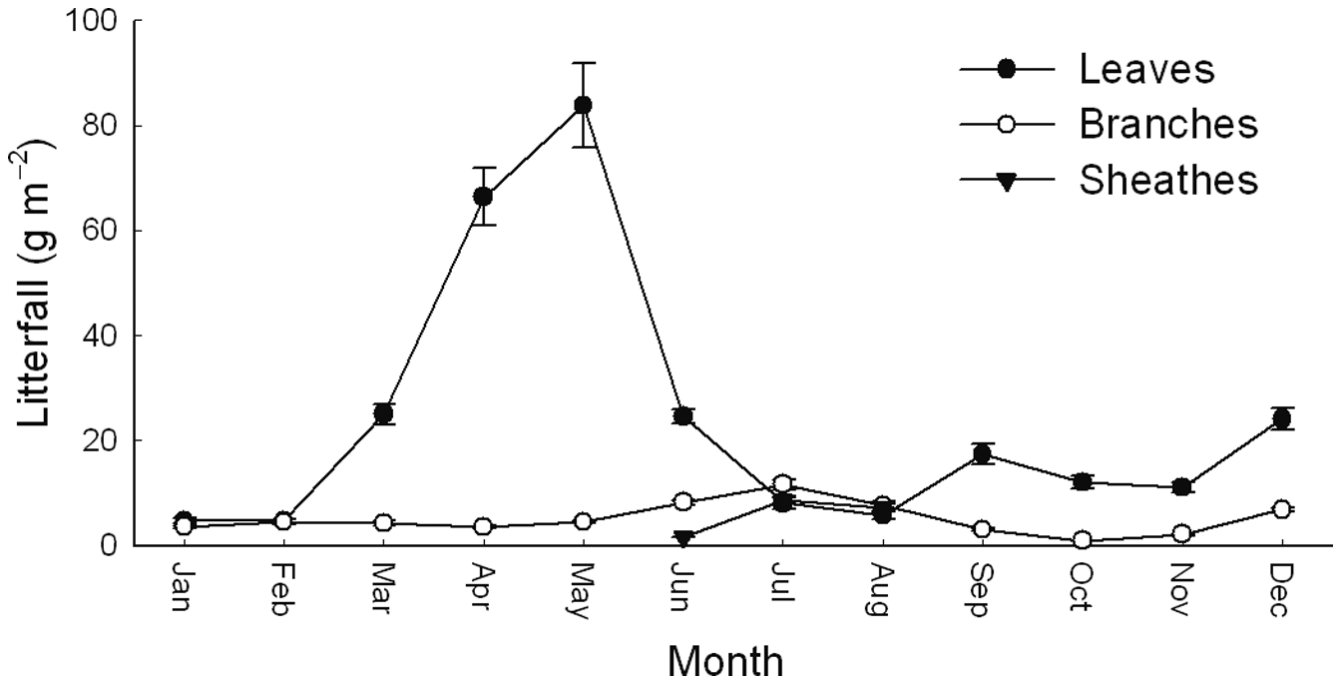
Monthly variations in litterfall fractions in a *Pleioblastus amarus* bamboo forest. Bars indicate ± SD, *n* = 10.

The estimated biomass of *P. amarus* was 157.0±4.7 Mg ha^−1^ (Table 1). The leaf litterfall peak occurred in May. Sheath litter only fell from June to August, and there was no significant seasonal variation in the branch litterfall (Figure 1). The mean litter production was 6.37 Mg ha^−1^. The N concentration for *P. amarus* leaves was 6.21 g N kg^−1^, which was higher than the other samples (0.65–3.56 g N kg^−1^). The total N in vegetation was 351.7±14.6 kg N ha^−1^, and the vast majority (85%) of the vegetation N pool was aboveground. The accumulation of litter was very low (2.0 Mg ha^−1^) and less than the annual litterfall input of 6.37 Mg ha^−1^. The N concentration in the soil decreased with the increase of soil depth (from 1.02 g kg^−1^ in the 0–5 cm soil horizon to 0.25 g kg^−1^ in the 80–100 cm soil horizon). The total N pool of soil (including the forest floor) was 7 752.8±458 kg ha^−1^, which accounts for 96% of all the N pools in this bamboo ecosystem.

**Table 1:** Concentrations, pools and fluxes of total nitrogen in vegetation by part, litterfall by litter fraction, and soils by horizon in a *Pleioblastus amarus* bamboo forest.

Vegetation	bole	branch	leaf	root wood	rhizome	root		TOTAL
Biomass (Mg ha^−1^)	80.4 (4.6)	20.0 (2.3)	17.1 (0.9)	10.6 (0.6)	23.6 (1.2)	5.3 (0.8)		157.0 (4.7)
N concentration (g kg^−1^)	1.42 (0.06)	3.56 (0.25)	6.21 (0.08)	0.65 (0.01)	1.55 (0.10)	1.94 (0.04)		
N pool (kg N ha^−1^)	114.1 (3.9)	77.7 (13.6)	106.0 (4.2)	6.9 (0.3)	36.6 (2.7)	10.4 (1.7)		351.7 (14.6)

Litterfall	leaf	branch	sheath					TOTAL
Litter production (Mg ha^−1^ yr^−1^)	5.37 (0.25)	0.48 (0.07)	0.52 (0.10)					6.37 (0.30)
N concentration (g kg^−1^)	3.88 (0.10)	3.53 (0.03)	5.19 (0.05)					
N flux (kg N ha^−1^ yr^−1^)	20.85 (1.29)	1.70 (0.29)	2.64 (0.09)					25.19 (1.51)

Soil horizon	forest floor	0–5 cm	5–20 cm	20–40 cm	40–60 cm	60–80 cm	80–100 cm	TOTAL
Bulk density (g cm^−3^)		1.15 (0.05)	1.27 (0.08)	1.40 (0.10)	1.48 (0.10)	1.50 (0.11)	1.52 (0.12)	
Total amount (Mg ha^−1^)	2.0 (0.16)	575 (25)	1905 (120)	2800 (200)	2960 (200)	3000 (225)	3040 (240)	
N concentration (g kg^−1^)	4.74 (0.32)	1.02 (0.04)	0.70 (0.05)	0.63 (0.01)	0.55 (0.01)	0.56 (0.04)	0.25 (0.01)	
N pool (kg N ha^−1^)	9.48 (0.79)	587 (41)	1330 (12)	1763 (115)	1627 (99)	1677 (122)	759 (53)	7752.8 (458)

SD in parentheses, Vegetation: *n* = 3, Litterfall: *n* = 10, Soil horizon: *n* = 3.

### Water flows

The incident PP recorded within the study area from January 1 to December 31, 2009, was 1984.2 mm (Table 2), a value higher than the annual mean PP of 1489.8 mm for the period 1980–2000. The mean amount of TF, SF, SR, FFL, SW1, and SW2 were 1663.9, 236.2, 14.2, 1072.3, 161.8 and 41.5 mm, respectively. Throughfall and SF were 83.9% and 11.9% of annual PP, respectively. Annual water flow input to the forest floor (TF+SF) was 1900.1 mm. The annual water flux input to mineral soil was 1072.3 mm (FFL). Surface runoff (14.2 mm) and SW2 (41.5 mm) was very low in this ecosystem.

**Table 2:** Monthly records of water flows from January to December 2009 in a *Pleioblastus amarus* bamboo forest.

Month	No. of rainy days	PP (mm)	TF (mm)	SF (mm)	SR (mm)	FFL (mm)	SW1 (mm)	SW2 (mm)
January	12	37.5 (3.0)	43.0 (3.3)	12.5 (0.8)	0.01 (0.00)	7.6 (0.6)	0	0
February	11	35.2 (2.5)	28.5 (2.0)	3.9 (0.2)	0.06 (0.01)	5.9 (0.5)	0	0
March	17	90.6 (7.6)	76.8 (7.1)	9.1 (0.6)	0.19 (0.01)	22.0 (1.2)	1.5 (0.1)	0.75 (0.04)
April	20	143.6 (9.0)	138.0 (10.3)	12.0 (0.1)	0.49 (0.04)	47.4 (3.0)	5.8 (0.4)	2.60 (0.21)
May	16	108.2 (8.0)	95.7 (7.4)	7.9 (0.1)	0.46 (0.04)	39.2 (2.5)	4.3 (0.3)	1.36 (0.10)
June	15	259.6 (13.0)	251.1 (11.1)	26.0 (0.1)	2.97 (0.20)	89.6 (6.0)	9.2 (0.6)	6.35 (0.35)
July	20	507.6 (23.0)	405.9 (19.8)	75.1 (0.5)	4.73 (0.36)	307.3 (12.0)	51.3 (2.3)	17.09 (1.00)
August	15	327.8 (2.8)	289.7 (3.0)	32.6 (0.2)	3.01 (0.25)	231.5 (10.0)	28.2 (1.2)	5.63 (0.23)
September	17	130.9 (7.6)	88.3 (6.1)	8.9 (0.1)	0.61 (0.05)	82.5 (5.0)	10.1 (0.7)	0.92 (0.07)
October	16	201.0 (7.0)	131.7 (4.3)	14.1 (0.2)	1.10 (0.09)	123.2 (8.9)	18.4 (1.1)	1.48 (0.10)
November	16	73.1 (5.3)	64.4 (3.0)	19.6 (0.2)	0.46 (0.04)	57.2 (4.5)	24.3 (1.0)	2.58 (0.12)
December	17	69.1 (4.6)	50.8 (2.2)	14.5 (0.1)	0.13 (0.01)	58.9 (3.5)	8.0 (0.7)	2.69 (0.11)
TOTAL	192	1984.2 (6.0)	1663.9 (5.9)	236.2 (9.9)	14.2 (0.3)	1072.3 (14.3)	161.8 (5.3)	41.5 (1.5)

Abbreviations: PP = precipitation, TF = throughfall, SF = stemflow, SR = surface runoff, FFL = forest floor leachate, SW1 = soil water at 40 cm belowground, SW2 = soil water at 100 cm belowground. SD in parentheses, *n* = 3.

The seasonal variations of water flows were similar. There were positive correlations among monthly PP, TF, SF, SR, FFL, SW1, and SW2 (*p*<0.05). Water fluxes in PP, TF, SF, SR, FFL, SW1, and SW2 from April to October accounted for 85%, 84%, 75%, 94%, 86%, 79% and 85% of annual fluxes, respectively.

### Nitrogen concentration in water flows

**Figure 2:**
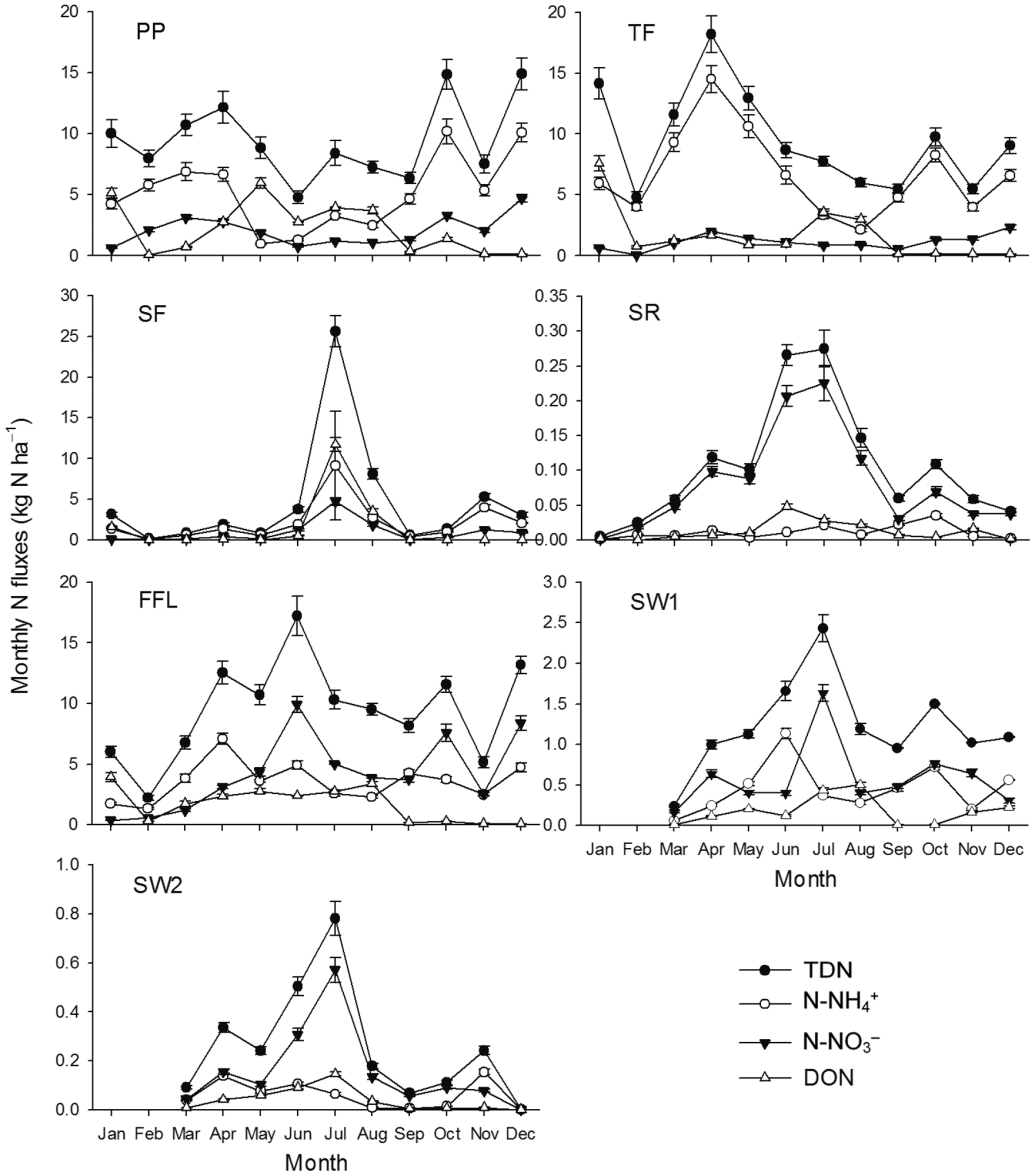
Seasonal variations of total dissolved nitrogen, N-NH_4_^+^, N-NO_3_^−^ and dissolved organic nitrogen concentrations in different flow paths. Abbreviations: TDN = total dissolved nitrogen, DON = dissolved organic nitrogen, PP = precipitation, TF = throughfall, SF = stemflow, SR = surface runoff, FFL = forest floor leachate, SW1 = soil water at 40 cm belowground, SW2 = soil water at 100 cm belowground. Bars indicate ± SD, *n* = 3.

**Figure 3:**
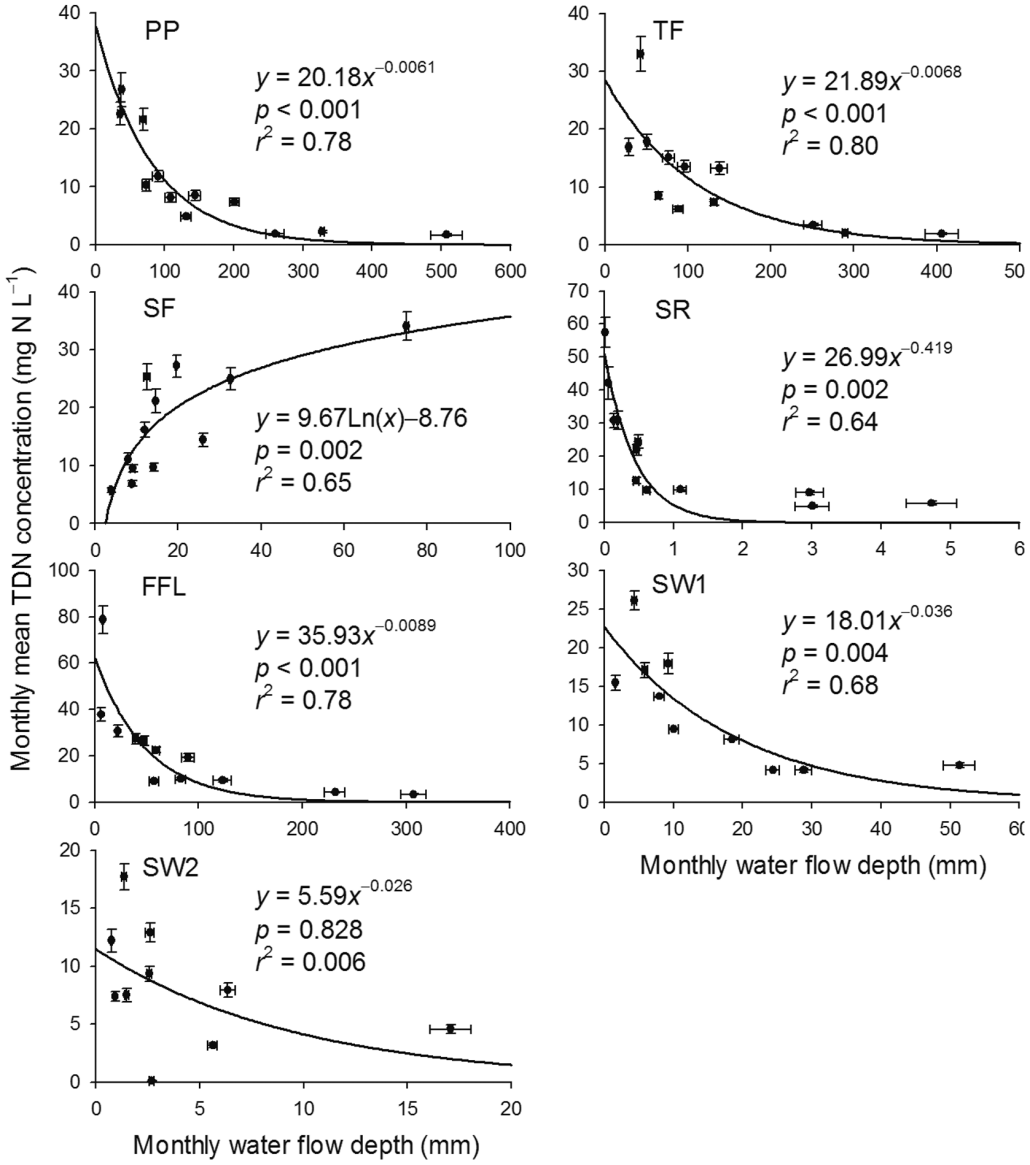
Relationships between monthly water flow depths and monthly mean total dissolved nitrogen concentrations. Abbreviations: TDN = total dissolved nitrogen, PP = precipitation, TF = throughfall, SF = stemflow, SR = surface runoff, FFL = forest floor leachate, SW1 = soil water at 40 cm belowground, SW2 = soil water at 100 cm belowground. Bars indicate ± SD, *n* = 3.

In general, the temporal concentration patterns of N-NH_4_^+^, N-NO_3_^−^ and DON were similar to that of TDN. Concentrations of TDN in PP, TF, SR and FFL followed a clear seasonal pattern, with the highest concentration in winter and the lowest in summer. While, since the precipitation is concentrated in summer, the fluxes (SF, SR, FFL, SW1 and SW2) of N species were higher in the summer (Figure 2). There were significant negative exponential relationships between monthly water flow depths and monthly mean TDN concentrations in PP, TF, SR, FFL and SW1 (Figure 3). Monthly TDN concentration in SF was logarithmically related to monthly water flow depth (Figure 3).

N-NH_4_^+^ was the dominant N form in PP, TF and SF solutions, suggesting that this was the major N form in atmospheric deposition. However, average annual volume-weighted concentrations of N-NO_3_^−^ in SR, FFL, SW1 and SW2 were higher than N-NH_4_^+^. Average annual volume-weighted concentrations of TDN in water flows decreased in the order FFL>SR>SW1>SF>TF>SW2>PP (Table 3). Annual mean concentrations of TDN, N-NH_4_^+^, N-NO_3_^−^ and DON were highest in FFL, TF, SR and FFL and lowest in SW2, SR, TF, and SW2, respectively.

**Table 3:** Annual mean concentrations (mg N l^−1^) of total dissolved nitrogen, N-NH_4_^+^, N-NO_3_^−^ and dissolved organic nitrogen in water flows in a *Pleioblastus amarus* bamboo forest.

Flow path	TDN	N-NH_4_^+^	N-NO_3_^−^	DON
PP	5.74 (0.32)	3.12 (0.15)	1.25 (0.06)	1.36 (0.08)
TF	6.84 (0.28)	4.81 (0.28)	0.82 (0.06)	1.21 (0.07)
SF	7.49 (0.51)	4.49 (0.24)	1.57 (0.08)	1.40 (0.06)
SR	9.15 (0.68)	0.70 (0.05)	7.04 (0.46)	1.41 (0.06)
FFL	10.56 (0.46)	3.96 (0.13)	4.72 (0.32)	1.87 (0.10)
SW1	7.54 (0.21)	2.84 (0.09)	3.58 (0.19)	1.11 (0.05)
SW2	6.27 (0.16)	1.45 (0.07)	3.61 (0.14)	0.96 (0.03)

Abbreviations: TDN = total dissolved nitrogen, DON = dissolved organic nitrogen, PP = precipitation, TF = throughfall, SF = stemflow, SR = surface runoff, FFL = forest floor leachate, SW1 = soil water at 40 cm belowground, SW2 = soil water at 100 cm belowground. SD in parentheses, *n* = 3.

The concentration of N-NH_4_^+^ in TF and SF was significantly higher than that in PP. The concentration of N-NO_3_^−^ in SF and TF were significantly higher and lower than that in PP, respectively. Concentrations of TDN, N-NH_4_^+^, N-NO_3_^−^ and DON decreased consistently with depth in the mineral soil, which changed from FFL to SW1 and SW2.

### Nitrogen fluxes through water flows

**Figure 4:**
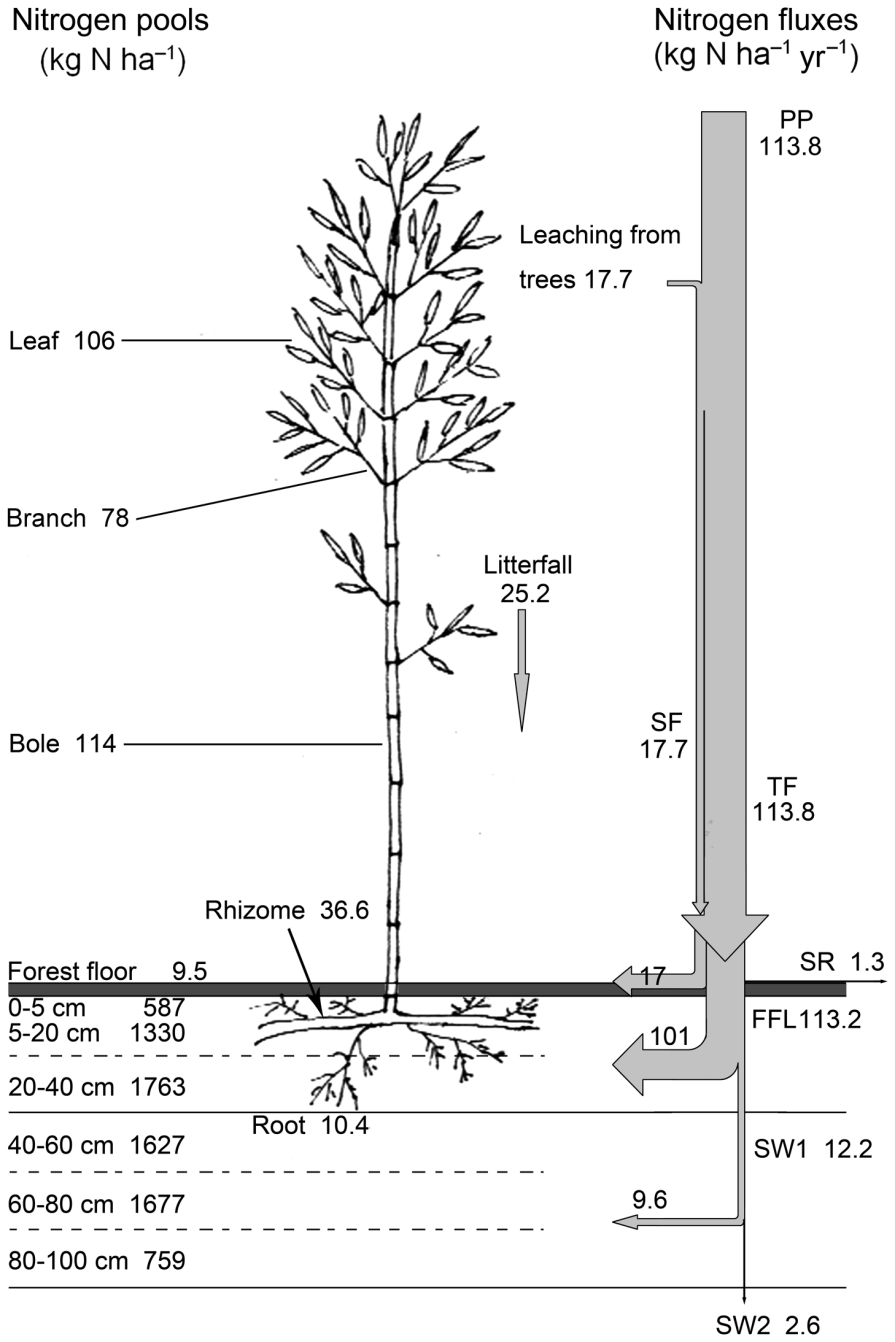
Pools and fluxes of nitrogen in a *Pleioblastus amarus* bamboo forest. Abbreviations: PP = precipitation, TF = throughfall, SF = stemflow, SR = surface runoff, FFL = forest floor leachate, SW1 = soil water at 40 cm belowground, SW2 = soil water at 100 cm belowground.

The bulk TDN deposition in PP at the study site was 113.8 kg N ha^−1^ yr^−1^. Dissolved inorganic nitrogen (DIN) contributed about 78% to the TDN deposition in this region and was mainly in the form of N-NH_4_^+^ (70% of DIN). Mean annual inputs of N-NH_4_^+^, N-NO_3_^−^ and DON in PP were 61.9, 24.9 and 26.9 kg N ha^−1^, and the total rain-based inputs to the forest floor (TF+SF) were 131.5, 90.7, 17.4 and 23.4 kg N ha^−1^ (Table 4; Figure 4). N-NH_4_^+^ was the dominant form of N input into the forest canopy (54%) and forest floor (69%). After FFL, N-NH_4_^+^ and N-NO_3_^−^ contributed 38% and 45% to TDN, respectively (Table 4).

**Table 4:** Annual fluxes (kg N ha^−1^ yr^−1^) as total dissolved nitrogen, N-NH_4_^+^, N-NO_3_^−^ and dissolved organic nitrogen and effects of forest vertical structure on N fluxes in a *Pleioblastus amarus* bamboo forest.

Flow path/Vertical effect	TDN	N-NH_4_^+^	N-NO_3_^−^	DON
PP	113.8 (8.5)	61.9 (1.1)	24.9 (0.3)	26.9 (0.5)
TF	113.8 (3.4)	80.1 (3.0)	13.7 (0.4)	20.1 (0.5)
SF	17.7 (0.3)	10.6 (0.4)	3.7 (0.2)	3.3 (0.2)
SR	1.3 (0.1)	0.1 (0.0)	1.0 (0.1)	0.2 (0.0)
FFL	113.2 (3.7)	42.5 (2.0)	50.6 (1.7)	20.1 (0.6)
SW1	12.2 (0.2)	4.6 (0.2)	5.8 (0.2)	1.8 (0.0)
SW2	2.6 (0.1)	0.6 (0.0)	1.5 (0.0)	0.4 (0.0)
input (PP)	113.8	61.9	24.9	26.9
Canopy effect (TF+SF–PP)	17.7	28.8	−7.5	−3.6
Litter effect (SR+LL–TF–SF)	−17.0	−48.0	34.2	−3.1
0–40 cm soil effect (SW1–LL)	−101.0	−38.0	−44.8	−18.3
40–100 cm soil effect (SW2–SW1)	−9.6	−3.9	−4.3	−1.4

Abbreviations: TDN = total dissolved nitrogen, DON = dissolved organic nitrogen, PP = precipitation, TF = throughfall, SF = stemflow, SR = surface runoff, FFL = forest floor leachate, SW1 = soil water at 40 cm belowground, SW2 = soil water at 100 cm belowground. SD in parentheses, *n* = 3. A positive value indicates the effect is increased, and a negative value indicates the decreased effect.

The annual N fluxes in PP, TF, SF, SR, FFL, SW1 and SW2 were 113.8, 17.7, 113.8, 1.3, 113.2, 12.2 and 2.6 kg N ha^−1^, respectively (Figure 4). The amount of TDN in SW1 was only 10.8% of that in FFL, suggesting that further TDN removal processes must be active in the rooting soil horizon (0–40 cm). The amount of TDN, N-NH_4_^+^, N-NO_3_^−^ and DON retained or be consumed by the 0–40 cm soil horizon was 89.2%, 89.2%, 88.5% and 91.0% of those entering the mineral soil, respectively.

## Discussion

### Nitrogen pools

Estimated biomass of *P. amarus* in this study was 157.0±4.7 Mg ha^−1^, which was comparable to another *P. amarus* plantation located in Zhejiang, eastern China (140.35 Mg ha^−1^ [30]), and similar to the mean value of *Phyllostachys pubescens* bamboo biomass in China (159.86 Mg ha^−1^ [33]). The amount of litterfall of this bamboo species (6.37 Mg ha^−1^ yr^−1^) was relatively close to a pure *Cunninghamia lanceolata* plantation (2.44–7.88 Mg ha^−1^ yr^−1^ [34]) and a mixed forest (4.45–10.41 Mg ha^−1^ yr^−1^ [34]) in the same climate region. The accumulation of litter was very low, less than the annual litterfall input. This result might be due to the fact that litter decomposes in this bamboo ecosystem very quickly [28].

### Water allocation in forest ecosystems

The temporal pattern of PP in the study site is due to the prevailing monsoon climate. Warm winds from the ocean causes copious amounts of PP during the summer monsoon season. In winter, dry continental air masses from Siberia result in a cold and dry climate. In the present study, seasonal variations of water fluxes in TF, SF, SR, FFL, SW1 and SW2 closely followed that in PP, with most of the water fluxes occurring during the warm season.

In this subtropical bamboo forest, the proportion of TF in annual PP was 83.9%, which was lower than that in tropical rain forests (92.4%–96.6% [20]), near the median value (89%) of the five subtropical forests reported by Chen and Mulder [16], and higher than the value in a moso bamboo (*Phyllostachys pubescens*) forest (74% [24]). Stemflow was very high in this bamboo ecosystem, accounting for 11.9% of annual PP. The proportion was higher than in tropical rain forests (1.5%–2.2% [20]; 2%–8% [21]). We may explain the high proportion of SF in PP by the following mechanisms. First, the branch surface of *P. amarus* is very smooth, so little water can be adsorbed. Second, the angle of the branches to the trunk is generally between 30° and 70°, so water can be funneled down along the trunk easily. Third, *P. amarus* is a small diameter, scattered bamboo species with a high trunk density, therefore, increasing the opportunity for the generation of stemflow. As a result, the forest canopy interception (*I*, *I* = PP−TF−SF) and I/PP ratio in this bamboo forest was very low (84.1 mm and 4.2%). Much of the PP was input to the forest floor (TF+SF = 1900.1 mm).

The water exchange mainly occurred in the litter floor layer and topsoil layer. The annual volume of water input to the forest floor was 1900.1 mm, whereas, annual water output through SR was very low (14.2 mm). Much of the water was evaporated to the atmosphere (about 600 mm, measured simply by us in the same site), and retained in the 0–40 cm soil horizon and the forest floor. The diameter of *P. amarus* roots were generally <2 mm and distributed in the 0–30 cm soil horizon. In the adjacent stand to this study, Tu et al. [35] reported that the fine root (<2 mm) biomass of the *P. amarus* plantation was 533 g m^−2^ in 0–30 cm soil horizon. Thus, a large part of the water in the soil may be taken up by fine roots for plant transpiration, growth and metabolism. The high density of fine roots also improved the soil structure, increased the porosity and enhanced the permeability of soil. So, the surface runoff was still very low even during heavy rains.

### Nitrogen deposition

The inorganic N concentration in our site was very high. In remote areas around the world, the concentration of N-NH_4_^+^ and N-NO_3_^−^ in precipitation ranges from 0.02–0.05 mg N l^−1^ and 0.02–0.08 mg N l^−1^ respectively [36]. Thus, the average annual volume-weighted concentrations of N-NH_4_^+^ (3.12 mg N l^−1^) and N-NO_3_^−^ (1.25 mg N l^−1^) in precipitation at our site were up to 62 and 16 times greater than values reported for remote sites, respectively. The concentration of inorganic N at our site (4.36 mg N l^−1^) was greater than the levels reported for urban areas in the western USA [37] and many areas of north and central Europe (e.g., van Leeuwen et al. [38]). It was also higher than the recently reported mean value (2.17 mg N l^−1^) observed from 2003 to 2005 in the Yangtze River Delta region [39]. Due to the high annual precipitation in the rainy zone of SW China, inorganic N deposition (86.8 kg N ha^−1^ yr^−1^) in our site was higher than those in the North China Plain (27 kg N ha^−1^ yr^−1^ [40]), the Yangtze River Delta region in eastern China (26.8 kg N ha^−1^ yr^−1^ [39]), the Jiangxi Province in southeastern China (70.7 kg N ha^−1^ yr^−1^ [41]), and the Guangzhou in southern China (52.5 kg N ha^−1^ yr^−1^ [42]). The average N deposition rate in mainland China in 2007 (13 kg N ha^−1^ yr^−1^ [43]) indicated serious N pollution.

Anthropogenic sources are thought to provide the most important contribution of inorganic N to precipitation [1]. The two main sources of anthropogenic reactive N are food production and energy production [1]. Accordingly, agricultural emission of NH_3_ and industrial emission of NO_x_ are the main sources of deposited N-NH_4_^+^ and N-NO_3_^−^ respectively. Therefore, the NH_4_^+^/NO_3_^−^ molar ratio in atmospheric wet deposition can be largely suggestive of the degree of industrialization [39], [42]. In the present study, NO_3_^−^ contributed less than NH_4_^+^, with an annual mean NH_4_^+^/NO_3_^−^ molar ratio with 2.5, to inorganic N in precipitation in 2009. This ratio indicates that although the annual N-NO_3_^−^ deposition from fossil fuel combustion in industry and transportation in the study site is very high (24.9 kg N ha^−1^ yr^−1^), N-NH_4_^+^ deposition from agriculture and human and animal excrement are much more significant in this region.

Both the concentration and the total amount of DON were very high at our site. Annual flux of DON through precipitation was 26.9 kg N ha^−1^ yr^−1^, which accounted for 23.6% of total wet N deposition. Although the proportion of DON in this study was slightly lower than the average value in China (28% [44]), the amount of DON deposition in our site was on the high side, even higher than the bulk N deposition in many areas in China. Zhang et al. [40] reported that DON in wet deposition averaged 8.6 kg N ha^−1^ yr^−1^ (30% of total N wet deposition) across 15 sites in China. On a global scale, DON deposition became an increasingly significant contribution (about 30%) to the total atmospheric N deposition [45], [46]. The major sources of atmospheric organic N includes byproducts of reactions between hydrocarbons and NO_x_, terrestrial sources of reduced (amino acids) N, the long-range transport of organic matter (dust, pollen, etc.) and bacteria [45]. The bioavailability of deposited DON is much the same as that of inorganic N, especially for N-poor systems [47].

We may explain the high level of N deposition in the study site by the following mechanisms. First, our site was located about 200 km southwest of Chengdu, a megacity with a population of more than 5 million. The well-known Chengdu Plain centers Chengdu City with about 30 million people and is an industrial and agricultural powerhouse, both historically and presently. The rapid development of industry and agriculture in recent decades in the Chengdu Plain makes a large amount of the reactive N emissions in the atmosphere in the region [48]. Second, the site of this study is located in the transitional zone of the Sichuan Basin to the western Sichuan plateau, and is under the direction of the prevailing wind from the Chengdu Plain. Orographic rain is very frequent in the study area due to the intense elevation change in the terrain. The annual precipitation during the study period was 1984.2, and the number of rainy days was 192. Therefore, in the study area, the majority of reactive N in the atmosphere could be collected by frequent precipitation.

### Nitrogen concentration and flux in water flows

In general, the temporal concentration patterns of N-NH_4_^+^, N-NO_3_^−^ and DON were similar to that of TDN, with the highest concentration in the winter and the lowest in the summer. A probable reason is that a small amount of incidental rainfall is sufficient in scavenging reactive N from the atmosphere, while subsequent precipitation within a short period only dilutes the previous precipitation [21]. It is supported by the data in this study (Figure 3), which shows that for PP there is a dilution of concentration in months with higher rainfall. The pattern for SF is very different, with SF concentrations increasing with volume. In general, TF or TF plus SF fluxes of N are often used as the total N deposition in forest ecosystems, and SF is often a better reflection of dry deposition than TF [14]–[17]. Therefore, the pattern in SF may be resulting from the significant mist deposition in monsoon season. Mist droplets often have very high concentrations of pollutants. In this region, the rainfall frequency is very high (number of rainy day is more than 200). So the air moisture in the forest is often saturated, especially in monsoon season. After rainfall, a part of the water vapor condensed in the leaves and fall on the ground (TF). Therefore, in some months the sum of TF and SF was higher than the value of PP. Average annual volume-weighted concentration of TDN increased in the order PP<TF<SF<SR<FFL, indicating water leaches TDN from the objects with which it comes in contact.

In the present study, we found concentrations of TDN increased significantly after water passed through the forest canopy (PP<SF<TF). Similar results have been reported in many different tropical rain forests [20], [22]. The increased TDN in SF and TF may be derived from leaching of N dry deposition absorbed by leaves, branches and trunks. The high level of N-NH_4_^+^ deposition (dry and wet) may induce higher N-NH_4_^+^ leaching from plant surface. As a result, canopy accumulated of N only occurred in the two kinds of N compounds (N-NO_3_^−^ and DON) in this bamboo ecosystem. Precipitation is not only an important source of nutrient input to the forest ecosystem, but it is also an important means of nutrient transfer from the canopy to the forest floor. For example, the rainfall loading of N accounted for 49%–79% and 31% of total N inputs to the forest floor in central African rainforest ecosystems [20] and Amazon rainforests [22], respectively. However, many studies suggested that a portion of TDN, especially N-NH_4_^+^ and N-NO_3_^−^, can be assimilated by forest canopies [49]. One study conducted in two Norway spruce (*Picea abies*) stands indicated that 41%–63% of atmospheric N deposition was retained in the canopy and could account for 8%–20% of the annual requirement [50]. Canopy assimilation could not be detected in this study.

Several studies (e.g. Butler and Likens [17]) demonstrated that in many forest ecosystems, total N deposition can be estimated by measuring N amounts in bulk net TF plus SF. According to this method, SF represents 13.5% of the total N deposition to the forest floor at our site. The proportion is similar to that in a deciduous forest ecosystem [17]. Annual inputs of TDN, N-NH_4_^+^, N-NO_3_^−^ and DON from the atmosphere and forest canopy to forest floor (TF+SF) were 131.5, 90.7, 17.4 and 23.4 kg N ha^−1^ yr^−1^, respectively. Fluxes of N-NH_4_^+^ decreased and fluxes of N-NO_3_^−^ increased as water moved from TF and SF to FFL. This difference reflects the high rate of net nitrification (transform N-NH_4_^+^ into N-NO_3_^−^) that occurs in the forest floor. The same phenomenon has been observed in an Amazon forest ecosystem [22].

N-NO_3_^−^ was the predominant form of N loss through SR. Although the concentration of TDN in SR was very high (9.15 mg N L^−1^), the concentration of N-NH_4_^+^ was only 0.70 mg N L^−1^. The probable reason is that soil colloids are usually negatively charged, so N-NO_3_^−^ can easily escape the adsorption by soil and moves with the water flow. This mechanism also partly explains why the concentration of N-NO_3_^−^ was always higher than N-NH_4_^+^ in FFL, SW1 and SW2.

Results in the present study indicated a large immobilization of N in the 0–40 cm soil horizon. Evidence for this comes from both the decline in concentration of N-NH_4_^+^, N-NO_3_^−^ and DON from FFL to SW1 and the vertical water movement from the litter layer to the under-rooting zone. We suggest that plant N demands may contribute to the concentration gradient and N lost from hydrologic flow paths in shallow soil because the fine roots in *P. amarus* forests are most abundant in the top 30 cm of the soil [35]. Chaves et al. [22] reported a large removal of N, mostly as N-NO_3_^−^, in hydrological flow paths leading from soil solution. Our previous study indicates that the net primary production in this bamboo forest was 21.9 Mg dry biomass ha^−1^ yr^−1^ [26]. Therefore, the N uptake and accumulated by plant was very high (48.18 kg N ha^−1^ yr^−1^). In general, culms of bamboo are connected by an extensive system of rhizomes, leading to rapid asexual reproduction of new culms. Culm density in the *P. amarus* plantation would increase over time. The growth rate would slow down and the N demand would decrease if there were no forest management, such as forest thinning. Volatilize ammonia and denitrification are two unknowns in the N balance in this site. The two processes may be important N loss from soil into atmosphere. In some forest systems with high rates of N deposition, gaseous losses of N are enhanced [51].

### Potential effect of N deposition

The growth requirement for the bamboo is 351.7(biomass N)/9 (years growth) = 39 kg N ha^−1^ yr^−1^, which is less than the annual input of reactive N (excluding DON) is 87 kg N ha^−1^ yr^−1^ through PP. However, the N output through volatilize ammonia and denitrification are unknown. So the supply of atmospheric N deposition for plant growth is not very clear. In an adjacent *P. amarus* stand, we conducted an N addition experiment (four levels of N treatments: 0, 50, 150, 300 kg N ha^−1^ yr^−1^). That study suggested that this *P. amarus* ecosystem is currently a C sink, N controls the primary production, and experimental N addition stimulates the C accumulation in the ecosystem by increasing the plant C pool [26]. In addition, the N concentration in the soil was so low (0.1% and 0.07% in 0–5 and 5–20 cm horizons, respectively) that it could not support the rapid plant growth. Most of the N in the soil was in the form of organic N, while the large organic N pool in the soil is not directly available for plant uptake. In the N addition experiment, we also found that microbial activities [26], extracellular enzyme activities [52], and the content of several fractions of active organic C [53] in the soil increased under the experimental N addition. Therefore, this ecosystem is N limited in its present state, and N inputs through precipitation and canopy transportation are very important for the soil fertility and plant growth. In a meta-analysis of 126 N addition experiments, LeBauer and Tresseder [7] reported that N limitation of net primary productivity in terrestrial ecosystems is globally distributed. However, continued high levels of N deposition may cause available N to exceed biological demands. Such a system is then considered N saturated and no longer has the capacity to assimilate additional N [54]. The “N saturation” concept describes cases in which N supply exceeds biotic demand, resulting in the alleviation of N limitation and distinctly nonlinear increases in DIN fluxes from soils to streams [54], [55]. Results from both theoretical and empirical studies have suggested that this occurs after thresholds of N loading and soil C∶N ratios are breached [56]–[58].The atmospheric N deposition in this area was extremely high. So it can be anticipated that this bamboo system and many other similar forest systems in this area would become N saturated systems within decades, under continuous increasing N deposition.

## Conclusion

The N input through total dissolved N (TDN) deposition in PP at the study site was 113.8 kg N ha^−1^ yr^−1^, which was on the highest level around the world. N-NH_4_^+^, N-NO_3_^−^ and DON accounted for 54%, 22% and 24% of total N deposition. Net canopy accumulated of N occurred with N-NO_3_^−^ and DON but not N-NH_4_^+^. Overall, forest canopy increased 17.7 kg N ha^−1^ yr^−1^ of the TDN flux input to the forest floor through TF and SF. The annual N fluxes in PP, TF, SF, SR, FFL, SW1 and SW2 were 113.8, 17.7, 113.8, 1.3, 113.2, 12.2 and 2.6 kg N ha^−1^, respectively. The water exchange and N consume mainly occurred in the litter floor layer and topsoil layer. Mean N pools in vegetation and soil were 351.7 and 7752.8 kg N ha^−1^. Annual mean N flux input to the forest floor through litterfall was 25 kg N ha^−1^ yr^−1^. The primary production is limited by N on the present state, high amount of N inputs through precipitation and canopy transportation are important for the soil fertility and plant growth. However, the N status of this bamboo system may be changing under the high level of atmospheric N deposition in the long run.
